# Autopsy and Cardiac Magnetic Resonance Image Case of Bevacizumab-Related Cardiomyopathy

**DOI:** 10.3390/jcdd9070208

**Published:** 2022-07-01

**Authors:** Naoki Hashimoto, Daisuke Kitano, Takehiro Tamaki, Yutaka Koyama, Akimasa Yamada, Kinta Hatakeyama, Hiroyuki Hao, Yasuo Okumura

**Affiliations:** 1 Division of Cardiology, Department of Medicine, Nihon University School of Medicine, Tokyo 173-8610, Japan; nh07261215@gmail.com (N.H.); takehiro.tamaki@gmail.com (T.T.); s4009539@gmail.com (Y.K.); akimasa09074033163@gmail.com (A.Y.); okumura.yasuo@nihon-u.ac.jp (Y.O.); 2 Division of Human Pathology, Department of Pathology and Microbiology, Nihon University School of Medicine, Tokyo 173-8610, Japan; 3 Department of Pathology, National Cerebral and Cardiovascular Center, Osaka 564-8565, Japan; kpathol@ncvc.go.jp

**Keywords:** bevacizumab, cardiomyopathy, congestive heart failure, cardiac magnetic resonance imaging, autopsy

## Abstract

We report an autopsy case of a 69-year-old female with cervical cancer. She was given bevacizumab-containing chemotherapy for 4 months. After two years of chemotherapy, she developed congestive heart failure (CHF) with left ventricular dysfunction. Cardiac magnetic resonance (CMR) imaging revealed late gadolinium enhancement (LGE) of linear mid-wall delayed enhancement located in the basal to the mid-septal wall, suggesting bevacizumab-related cardiotoxicity. Although she was treated with cardioprotective medications and discharged, she eventually died from worsening CHF a year later, and we conducted an autopsy. Histopathological examination revealed diffuse fibrosis in the myocardium, and the area where LGE was present on CMR showed thinning and wavy changes in cardiomyocytes with diffuse interstitial fibrosis and edema.

## 1. Introduction

Cancer treatment at present employs a combination of chemotherapy, radiotherapy, and surgery to prolong life and provide a cure. However, many of these treatments can cause cardiovascular complications such as heart failure, hypertension, thromboembolism, myocardial ischemia, and arrhythmias [1]. These cardiotoxicities related to anticancer treatment can interfere with the efficiency of treatment, decrease quality of life, or impact the actual survival of the patient with cancer, so they are likely to remain a significant challenge for both cardiologists and oncologists. Though bevacizumab has been approved for treating several kinds of cancers, some cardiotoxicities are reported as side effects [2].

## 2. Case Presentation

A 69-year-old woman with cervical cancer presented with acute decompensated heart failure. Two years before heart failure development, she was diagnosed with cervical cancer and underwent chemotherapy combined with bevacizumab (4.9 mg/kg/week) and paclitaxel (1.0 mg/kg/week) for 4 months. Before chemotherapy, she had no history of cardiovascular disease including hypertension; electrocardiography (ECG) showed a sinus rhythm and narrow QRS morphology with nonspecific T wave abnormalities (Supplemental Figure S1A), and echocardiography showed a left ventricular end-diastolic dimension (LVDd) of 53 mm and LV ejection fraction (LVEF) of 47% (Supplemental Figure S1C). After starting the chemotherapy, her systolic blood pressure increased from 100–130 mmHg to 150–170 mmHg, and the blood pressure promptly returned to initial levels after the completion of 4-months chemotherapy.

**Figure 1:**
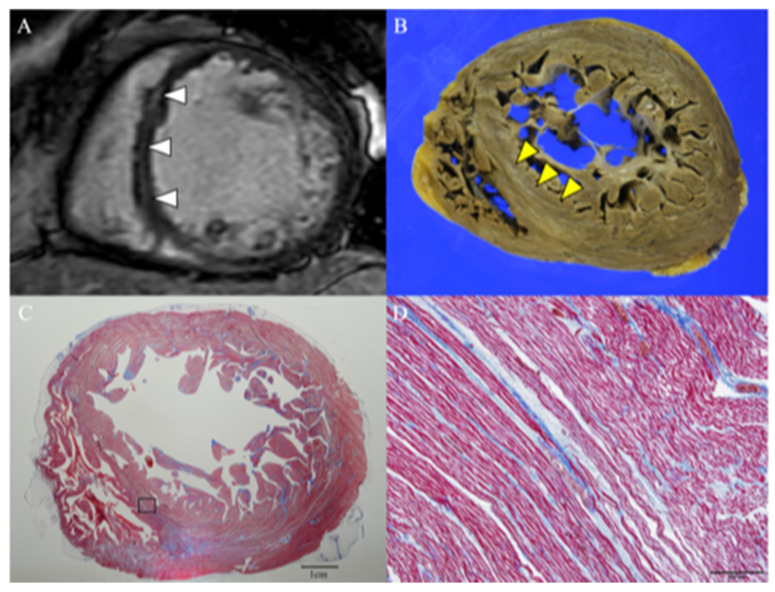
Cardiac Magnetic Resonance Imaging and Histopathological Findings. Cardiac magnetic resonance (CMR) short-axis image of the mid-ventricle revealed a linear late gadolinium enhancement (LGE) of the septal wall (arrow) (**A**). Macroscopic autopsy image corresponding to the CMR image showed fibrosis in the mid-ventricle septal wall (arrow) (**B**). Loup image showed diffuse fibrosis (blue) in the Masson’s trichrome stained ventricular myocardium sample (**C**). Magnitude image (×100) from a region of LGE in the above CMR image demonstrated a thinning and wavy change in cardiomyocytes and massive interstitial fibrosis and edema (**D**).

On admission, her breathing was rapid, and she had tachycardia, jugular venous distension, and lower-extremity edema. Her blood pressure was 96/59 mmHg, with a heart rate of 125/min. ECG demonstrated sinus tachycardia with a wide QRS complex in the pattern of left bundle branch block (Supplemental Figure S1B). Chest radiography revealed enlarged cardiac silhouette and pulmonary congestion. Serum cardiac troponin and creatine kinase levels were negative, and N-terminal pro-brain natriuretic peptide levels were elevated to 22,933 pg/mL. Echocardiography revealed global left ventricular dysfunction, with an LVEF of 17% and severe dilatation of the left ventricle (LVDd, 71 mm) (Supplemental Figure S1D). Myocardial scintigraphy results were normal. CMR imaging revealed a linear late gadolinium enhancement (LGE) in the mid-wall, located from the basal to the mid-septal wall (Figure 1A), with increased extracellular volume fraction (ECV) (35%). Given the recent use of bevacizumab and the cardiac imaging findings, bevacizumab-related cardiotoxicity was considered. Although the patient was given cardioprotective medications (carvedilol, candesartan, eplerenone, dapagliflozin, and ivabradine) and discharged, the LV enlargement and LV systolic dysfunction did not improve, and she eventually died from worsening heart failure triggered by a urinary tract infection one year later. An autopsy revealed a heart weight of 800 g, with no hypertrophy but diffuse fibrosis in the LV wall; that in the mid-ventricle septal wall was significant in terms of gross pathology (Figure 1B). No evidence of coronary artery or valvular disease was identified. Histological examination demonstrated diffuse interstitial fibrosis in the myocardium (Figure 1C), and a magnified image of the area in which LGE was present in CMR imaging showed a thinning and wavy change in cardiomyocytes with diffuse interstitial fibrosis and edema (Figure 1D).

## 3. Discussion

Bevacizumab, a humanized monoclonal immunoglobulin G_1_ antibody that targets vascular endothelial growth factor (VEGF), is widely used for the treatment of various solid tumors. Following the rapid introduction of VEGF signaling inhibitors (VEGFIs), a range of VEGFIs-associated cardiovascular toxicities has been reported. VEGFIs-associated hypertension has received the most attention, with an increase in blood pressure occurring in 80–90% of patients treated with VEGFIs. Moreover, VEGFIs are associated with a wider range of cardiovascular toxic effects, including left ventricular systolic dysfunction (LVSD), heart failure, arterial and venous thromboembolism, QT interval prolongation, and arrhythmia. The incidence of VEGFIs-associated LVSD may range from 10 to 20%, with the incidence of heart failure being approximately 1–5% [3]. Although the pathophysiological mechanisms underlying VEGFI-associated cardiotoxicity has been poorly defined, both the direct cardiomyocyte toxic effects of VEGFI and the indirect effects of uncontrolled hypertension leading to LV hypertrophy are considered the main mechanism [4]. VEGF induces the diastole of an endothelium-dependent coronary artery by stimulating endothelial cells to release nitrous oxide and prostacyclin, thereby relieving hypertension. As a result, by inhibiting VEGF, bevacizumab can lead to vasoconstriction and hypertension. It has been demonstrated that VEGFIs induce pressure overload through the reduction in myocardial capillary density, global contractile dysfunction, and cardiac fibrosis, eventually contributing to cardiac hypertrophy and heart failure [5]. However, the patient reported here had no history of hypertension or no evidence of hypertension and the increase in blood pressure with bevacizumab was temporary. At the time when the cardiac dysfunction occurred, there were no findings of left ventricular hypertrophy on cardiovascular imaging modalities. The autopsy revealed LV dilation rather than LV wall thickness, and histological examination revealed the thinning and wavy change in cardiomyocytes with diffuse interstitial fibrosis and edema. These findings may indicate not a response to a chronic stimulus due to bevacizumab-induced hypertension. Moreover, in this case, she had mild systolic dysfunction before chemotherapy. CMR imaging demonstrated increased ECV and linear LGE in the mid-wall located from the basal to the mid-septal wall, which may be a typical distribution and location of non-ischemic cardiomyopathies such as dilated cardiomyopathy or drug-induced cardiomyopathy [6,7]. However, as mentioned above, no history or cardiovascular imaging findings suggested dilated cardiomyopathy or other cardiovascular diseases. In particular, histological examination showed irreversible changes in cardiomyocytes due to direct cardiac injury; the severe degeneration of cardiomyocytes was similar to that after anthracycline treatment [8]. Therefore, the cardiac dysfunction, in this case, was considered to be due to direct bevacizumab-related cardiotoxicity. In other words, considering this case, the presence of linear LGE in the mid-wall of the septum on CMR imaging after bevacizumab treatment may indicate irreversible myocardial injury caused by bevacizumab. The easy and sensitive detection of LV fibrosis and dysfunction using CMR imaging may help in the early diagnosis of bevacizumab-related cardiomyopathy. Furthermore, if LGE is observed in CMR imaging, this means that the myocardium is strongly damaged and cardioprotective drugs should be administrated and continued for as long as possible. Therefore, future studies are required to validate the identification of positive delayed gadolinium enhancement using CMR imaging as a subclinical biomarker for future LV dysfunction. Here, we report the first case demonstrating a correlation between bevacizumab-associated histological fibrosis and LGE in CMR imaging.

A limitation of this case is that the patient had mild LV dysfunction before chemotherapy. The possibility that the patient had the organic cardiac disease before the chemotherapy cannot be completely ruled out. However, the patients certainly had no history of cardiovascular disease including hypertension and no cardiovascular family history and there was no evidence of LV hypertrophy, LV enlargement, LV asynergy, or valvular disease before chemotherapy. Considering the above and pathohistological findings, it is unlikely that organic myocardial injury had originally been present before chemotherapy.

## 4. Conclusions

This is the first case report of bevacizumab-related cardiotoxicity based on CMR imaging and histopathological findings. The early detection of LV dysfunction using CMR-LGE may allow for one to adjust treatment with bevacizumab or cardioprotective treatment before the development of irreversible heart failure.

## Data Availability

Not applicable.

## Supplementary Materials

The following supporting information can be downloaded at: https://www.mdpi.com/article/10.3390/jcdd9070208/s1, Figure S1: Changes in electrocardiogram and echocardiogram before and after bevacizumab treatment.
