# Supplementary material for: Autopsy and Cardiac Magnetic Resonance Image Case of Bevacizumab-Related Cardiomyopathy
Source: J Cardiovasc Dev Dis. 2022 Jul 1;9(7):208. doi: 10.3390/jcdd9070208 (PMC9318594; doi:10.3390/jcdd9070208)
Supplement: Supplementary file 1 [file jcdd-09-00208-s001.zip › jcdd-1737939-supplementary.pdf]

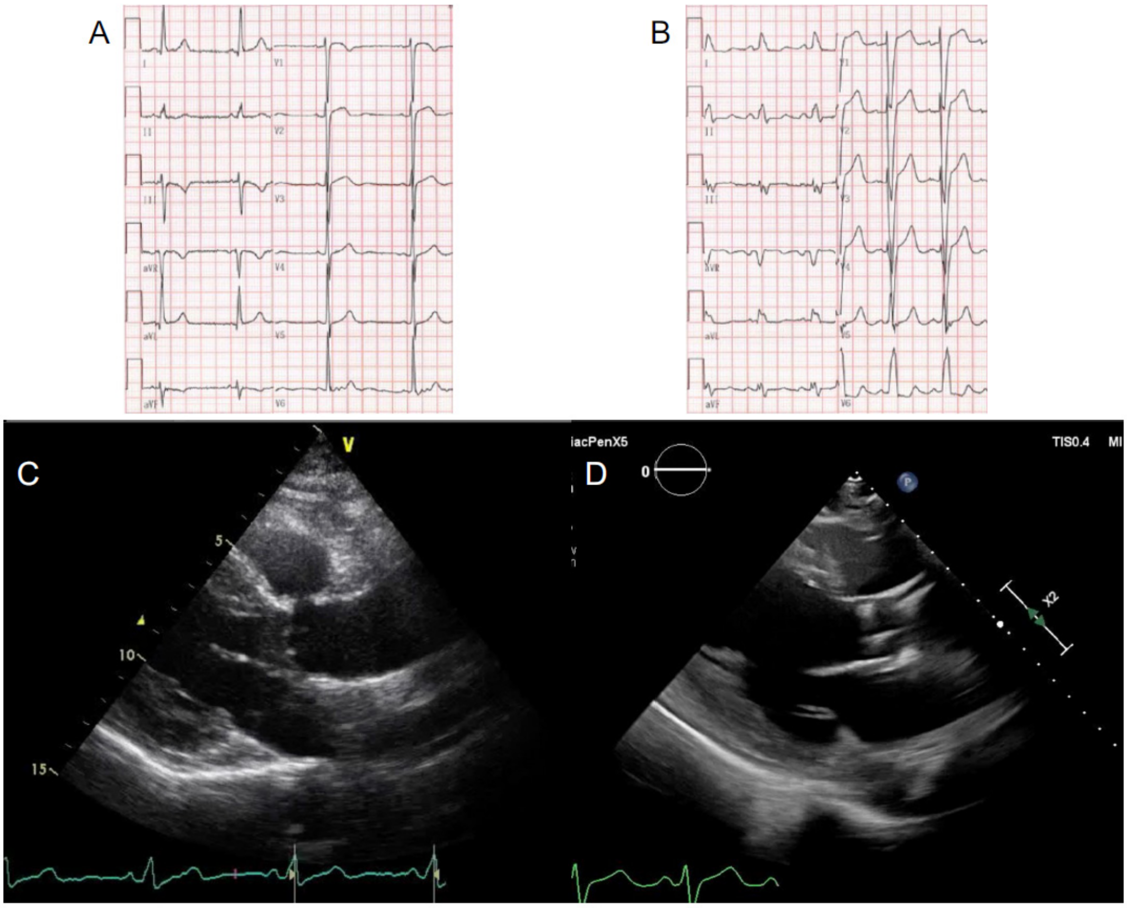


**Figure S1.** Changes in electrocardiogram and echocardiogram before and after bevacizumab treatment. Although an electrocardiogram (ECG) before chemotherapy showed only nonspecific T-wave abnormality (**A**), the ECG at the development of heart failure changed to a left bundle branch block pattern (**B**). On the other hand, an echocardiogram before chemotherapy showed mild left ventricular (LV) systolic dysfunction (LV ejection fraction 47%) (**C**), but the echocardiogram at the development of heart failure showed LV enlargement (LV end-diastolic dimension 71 mm) and severe LV systolic dysfunction (LV ejection fraction 17%) (**D**).
